# Integrated field control of *Dactylopius opuntiae* (Cockerell) (Hemiptera: Dactylopiidae) in *Opuntia* spp. using biorational methods

**DOI:** 10.3389/fmicb.2025.1714087

**Published:** 2025-11-24

**Authors:** Mohamed El Aalaoui, Said Rammali, Fatima Zahra Kamal, Alin Ciobica, Cristina Albert, Vasile Burlui, Bogdan Novac, Bouchaib Bencharki, Mohamed Sbaghi

**Affiliations:** 1 National Institute of Agricultural Research, Rabat, Morocco; 2 Human Nutrition, Bioactives and Oncogenetics Team, Faculty of Sciences, Moulay Ismail University, Meknes, Morocco; 3 Laboratory of Agro-Alimentary and Health, Faculty of Sciences and Techniques, Hassan First University of Settat, Settat, Morocco; 4 Health Care and Biology-Health Team, 2S2D Laboratory, Higher Institute of Nursing and Health Techniques of Casablanca (ISPITS), Casablanca, Morocco; 5 Department of Biology, Faculty of Biology, “Alexandru Ioan Cuza” University of Iasi, Iasi, Romania; 6 “Olga Necrasov” Center, Department of Biomedical Research, Romanian Academy, Iasi, Romania; 7 “Ioan Haulica” Institute, Apollonia University, Iasi, Romania; 8 CENEMED Platform for Interdisciplinary Research, “Grigore T. Popa” University of Medicine and Pharmacy of Iasi, Iasi, Romania; 9 Clinical Department, Apollonia University, Iasi, Romania; 10 Faculty of Medicine, “Grigore T. Popa” University of Medicine and Pharmacy of Iasi, Iasi, Romania

**Keywords:** biological control, integrated pest management (IPM), biorational insecticides, entomopathogenic fungi, cactus pear pest management, sustainable agriculture

## Abstract

**Introduction:**

*Dactylopius opuntiae* (Cockerell) is a major pest affecting *Opuntia* spp. plantations, causing significant economic losses. The study was conducted at the experimental domain of the National Institute of Agricultural Research, Zemamra, Morocco from 2022 to 2024.

**Objective:**

This study aimed to evaluate the efficacy of 19 treatments, including biorational insecticides—mineral oil (MO), black soap (BS), *Nicotiana glauca* extract (NG)—and the entomopathogenic fungus *Alternaria murispora* (AM), applied alone or in combinations, with or without pruning, in a randomized complete block design as integrated pest management strategies for *D. opuntiae*.

**Methods:**

Treatments were applied eight times per year at five-week intervals. Data collected included fruit number, cladode number, fruit weight, fruit shape index, phenological phases, infestation level, and visual quality of treated plants.

**Results:**

The results showed that the combined treatment MO + BS + NG + AM, particularly with pruning, significantly enhanced fruit production (51.1–113.3 across the 3 years), cladode production (26.5–71.4 across the 3 years), fruit weight (188.2–192.7 g across the 3 years), and visual quality of treated plants (visual quality scores: 9.8–10.0 across the 3 years). It also reduced fruit maturation duration (60.9–59.3 days across the 3 years), fruit development period (122.3–117.1 days across the 3 years), and *D. opuntiae* infestation (scale values: 0.2–0.0 across the 3 years). Pruning played a crucial role in improving the effectiveness of treatments by facilitating coverage and enhancing plant health.

**Conclusion:**

This study demonstrates that combining pruning with biological and botanical methods sustainably boosts *Opuntia ficus-indica* growth and controls *D. opuntiae*.

## 1 Introduction

Arthropod pests cause about 20% of global crop losses, estimated at over US$470 billion (Culliney, 2014). Recent data indicate that invasive pests and pathogens lead to up to 40% yield losses annually, with economic impacts exceeding US$220 billion (Mihale et al., 2009; FAO, 2021; CABI, 2022). Climate change further exacerbates pest problems by accelerating their development and range expansion (Deutsch et al., 2018). The cochineal pest *Dactylopius opuntiae* (Cockerell) (Hemiptera: Dactylopiidae) is one of the most destructive pests of *Opuntia* spp. worldwide (Sabbahi and Hock, 2022; Mendel et al., 2024). Since its outbreak in Morocco in 2014, it has devastated nearly 160,000 hectares of plantations, causing severe economic and ecological damage (Sbaghi et al., 2019). Similar destruction has been reported in Brazil, Tunisia, and Algeria (Lopes et al., 2010; Mazzeo et al., 2019; García Pascual et al., 2024). The pest attacks all parts of the plant, and infestations covering 75% or more of the cladode surface can lead to plant death within 3 months (Flores-Hernández et al., 2006; Vanegas-Rico et al., 2015; El Aalaoui et al., 2020). Chemical control remains the most commonly used strategy against *D. opuntiae* worldwide; however, its effectiveness is limited by the pest’s waxy coating, high fecundity (over 400 eggs per female), and development of insecticide resistance (Badii and Flores, 2001; Vanegas-Rico et al., 2015). In Morocco, pyriproxyfen and chlorpyrifos are frequently used but often require repeated applications, leading to residue contamination and environmental harm (El Aalaoui et al., 2019; Gitzel et al., 2024). Therefore, sustainable and environmentally safe control alternatives are urgently needed. In many countries where *D. opuntiae* has caused significant damage, integrated pest management (IPM) programs have been developed, combining multiple control methods with a focus on eco-friendly and environmentally sustainable solutions. These include the use of biorational insecticides, entomopathogenic microorganisms, and botanical extracts (da Silva Santos et al., 2016; Mazzeo et al., 2019) to provide eco-friendly and sustainable solutions, with no risk to non-target species or the environment. These approaches aim to manage pest populations effectively while minimizing harm to non-target organisms and promoting long-term ecological balance (Pratheeba et al., 2019; Vivekanandhan et al., 2024a,b, 2025a,b).

Biorational insecticides have been widely and, in some cases, successfully used to control *D. opuntiae* infestations under field conditions (Bouharroud et al., 2018; El Aalaoui et al., 2019). Mineral oil (780 g/L) at 2400 cc/hL demonstrated high efficacy against both nymphs and adult females, with 91.94% mortality recorded 120 h after treatment under field conditions (El Aalaoui et al., 2019). Under field conditions, black soap (60 g/L) applied in combination with *Capsicum annuum* (chili pepper) (Solanaceae) macerated extract (200 g/L) resulted in the highest mortality rates, with 87.31% mortality of adult females at 168 h post-application and 84.9% mortality of nymphs at 72 h post-treatment, with second application of black soap (60 g/L) at a three-day interval significantly increased adult female mortality, reaching 82.5% 3 days after the second treatment (Ramdani et al., 2021).

Entomopathogenic fungi (EPF) can kill insects at multiple life stages due to their persistence (Gul et al., 2014). Several isolates have been evaluated against *D. opuntiae*, showing potential for biological control (Torres and Giorgi, 2018). The *Fusarium incarnatum–equiseti* species complex (FIESC) was effective in Brazil (Carneiro-Leão et al., 2017). In Mexico, *Beauveria bassiana* (Balsamo) (Hypocreales: Cordycipitaceae), *Metarhizium anisopliae* (Metschn.) (Hypocreales: Clavicipitaceae), and *Verticillium lecanii* (Zimm.) (Hyphomycetes: Moniliales) caused high mortality of nymphs, with *M. anisopliae* and *V. lecanii* being the most effective (Ramírez-Sánchez et al., 2019). Under laboratory and greenhouse conditions, *B. bassiana*, *Akanthomyces lecanii* (Zimm.) Spatafora, Kepler & B. Shrestha (Hypocreales: Cordycipitaceae), and *Cordyceps farinosa* (Holmsk.) Kepler, Shrestha & Spatafora (Hypocreales: Cordycipitaceae) caused high mortality of nymphs and young females. The HASS strain of *B. bassiana* achieved 100% mortality at 10^8^ conidia/mL (Ramdani et al., 2022). Additionally, *Ulocladium* spp. and *Alternaria* spp. caused 70–90% mortality under laboratory conditions at 25 °C (Ouguas et al., 2022). In Morocco, *Alternaria murispora* (PP264308) and *A. destruens* (PP264311) (Pleosporales: Pleosporaceae) produced 84.2 and 75.8% mortality of adult females, respectively, under field conditions (El Aalaoui et al., 2024a). *Alternaria murispora* (PP264308) was also effective against *Phenacoccus solenopsis* Tinsley (Hemiptera: Pseudococcidae) in both laboratory and field studies (El Aalaoui et al., 2024c). It is worth noting that *A. murispora* functions not only as an entomopathogen but also as a foliar endophyte in olive trees, demonstrating ecological versatility (Nicoletti et al., 2020). However, its use as a biopesticide requires caution due to the potential of *Alternaria* spp. to produce mycotoxins (Kaur et al., 2019), which, although usually present at low levels in foods and crops, lack specific regulatory standards (Chen et al., 2021). Further studies on the metabolic profile of *A. murispora* are needed to ensure safe agricultural application.

Several studies have demonstrated the potential for controlling *D. opuntiae* using plant extracts under field conditions (da Silva Santos et al., 2016; Ramdani et al., 2021; Zim et al., 2024). Macerated extracts of *Capsicum annuum* (chili pepper), *Nicotiana glauca* (tree tobacco) (Solanaceae), and *Ricinus communis* (castor bean) (Euphorbiaceae) at concentrations of 8–10% have shown high efficacy against *D. opuntiae* under both laboratory and field conditions, and these plants are widely available in Moroccan ecosystems (Ramdani et al., 2021; Zim et al., 2024). The primary bioactive compounds responsible for their pesticidal effects include capsaicin and dihydrocapsaicin in *C. annuum* (Zewdie and Bosland, 2001), pyridine alkaloids such as nicotine and anabasine in *N. glauca* (Ali Alghamdi, 2021; Zim et al., 2024), and ricin (a toxic protein) and ricinine (an alkaloid) in *R. communis* (Elijah and Somadina, 2020). There are very few published studies on the use of combined methods to control *D. opuntiae* under field conditions. The use of *Fusarium* spp. isolates obtained from *D. opuntiae* collected in the field, combined with a 5% aqueous extract of *R. communis* (da Silva Santos et al., 2016) and *Enterolobium contortisiliquum* Vell. Morong (Fabaceae) (Velez et al., 2019), has proven to be a promising method for controlling *D. opuntiae*. In Brazil, isolates of *Fusarium caatingaense* (Sordariomycetes: Hypocreales) have been used to control *D. opuntiae*, either alone or in combination with aqueous and hydroethanolic extracts of *Nicotiana tabacum* (Solanaceae) and *Paubrasilia echinata* (Fabaceae), with promising results (Gonçalves Diniz et al., 2020).

At high infestation levels, pruning is one of the fundamental components of IPM programs against *D. opuntiae*. Pruning helps manage *D. opuntiae* by removing infested cladodes, reducing pest populations, and improving the effectiveness of applied treatments. However, tree pruning may reduce potential yield by removing fruit-bearing cladodes. Nevertheless, increased sunlight and air circulation can enhance fruit growth on the remaining wood, leading to either a positive or negative net effect on yield (Lefebvre et al., 2015). Additionally, the plant’s phenological cycle must always be considered when planning pruning activities. Understanding key stages of *Opuntia* spp.’s phenological cycle, such as the emission of vegetative and floral buds and fruit development, is essential (Matias et al., 2023). Despite the known benefits of pruning, few studies have evaluated its combined effects with biorational insecticides, entomopathogenic fungi, and plant extracts under field conditions. Therefore, the objective of this study was to assess the field efficacy of these integrated strategies for managing *D. opuntiae* infestations.

## 2 Materials and methods

### 2.1 *Dactylopius opuntiae* culture

*Dactylopius opuntiae* used in this study were sourced from a colony reared on *Opuntia ficus-indica* (L.) Mill. The insects were kept in mesh-covered wooden cages (50 × 50 × 50 cm) within an environmental chamber at the insectarium of the National Institute of Agricultural Research (INRA), Zemamra- Morocco (32°37′48″N, 8°42′0″W, Elevation 165 m). Rearing conditions were 25 ± 2 °C and 60 ± 10% RH, with a 12:12 h light–dark cycle. The colony was initially established from infested *Opuntia* spp. cladodes collected from Zemamra fields (El Aalaoui et al., 2019; El Aalaoui and Sbaghi, 2022). To enhance the population and monitor insect age, the “cladode-cutting technique” (Aldama-Aguilera and Llanderal-Cázares, 2003) was used. Mature cladodes were trimmed to uniform size, perforated at the base with a wooden stake, scarified for 48 h, and then hung vertically from metal grids in the cages.

### 2.2 Fungal isolate

The *Alternaria murispora* isolate (NCBI GenBank Acc. No: PP264308) used in this study was obtained from the INRA insectarium in Zemamra. This isolate was originally isolated from sterilized cadavers of *D. opuntiae* (Moroccan biotype), identified based on spore and colony morphology, and confirmed through ITS sequencing (El Aalaoui et al., 2024a).

The fungus was revived from Potato Dextrose Agar (PDA) (Biokar Diagnostics, France) stored at −80 °C and grown on 90-mm PDA Petri dishes using 15 mL of PDA for 20 days at 24–29 °C in darkness (Hernández-Valencia et al., 2024). Conidia and mycelia were scraped using a sterile scalpel, mixed with 20 mL of 0.03% Tween 80 solution in a 50-mL sterile centrifuge tube, vortexed for 5 min, and filtered to remove mycelial fragments. The conidial concentration was adjusted to 1.0 × 10^8^ conidia mL^−1^ using a hemocytometer (HGB, Germany), which corresponds to the recommended field application rate (El Aalaoui et al., 2024a,b,c). Conidial viability, assessed following Inglis et al. (2012), was consistently above 98%. Suspensions were stored at 4 °C and used within 12 h.

### 2.3 Plant extract

The leaf extract of *N. glauca*, known for its insecticidal properties, was prepared at the INRA insectarium following the method outlined by Ramdani et al. (2021). Leaves were collected from fields in Zemamra (32°37′48″N, 8°42′0″W; Elevation 165 m). They were washed and then dried at 26 ± 2 °C with 66 ± 5% RH under a 16:8 h light/dark cycle for 5 days. Drying continued until the leaves reached a constant weight. The dried leaves were then ground into a fine powder using an electric blender. For extraction, 100 g of powdered leaves was soaked in 1 L of distilled water for 72 h. The mixture was filtered through Whatman No. 1 paper, and the liquid extract was collected in a round-bottom flask. It was then concentrated using a Martin Christ Gefriertrocknungsanlagen GmbH freeze-dryer and stored at 4 °C. The extract was tested at the recommended field application rate (10%) for *D. opuntiae* control (Ramdani et al., 2021; Zim et al., 2024).

### 2.4 Biorational insecticides

Insecticide 101 (780 g mineral oil/L) (UPL, Ankleshwar, India) at 2400 cc/hL and Moroccan black soap at 60 g/L, purchased from the local market in Zemamra, were tested against *D. opuntiae*. The tested doses followed the recommended field application rates from previous studies (El Aalaoui et al., 2019; Ramdani et al., 2021).

### 2.5 Study site and period

The study was conducted at the experimental domain of the National Institute of Agricultural Research (CRRA SETTAT-INRA, Morocco) in Zemamra, located in the Casablanca-Settat region (33°15’ N, 8°30’ W). This site is situated in a semi-arid ecological zone, with an average annual rainfall of 330 mm over the past 30 years (1994–2023). Temperatures range from −1 °C (December–January) to 40–45 °C (July–August).

The soil at the site is a vertisol with an angular structure in the top 15 cm and extends to a depth of 1.5 meters. It is difficult to work with in dry conditions but becomes more manageable when moisture content is high. The soil has an alkaline pH, and its chemical composition prior to the study was as follows: nitrogen (200 mg/kg), phosphorus (P2O5, 46 mg/kg), potassium (K2O, 203 mg/kg), molybdenum (Mo, 1.5 mg/kg), electrical conductivity (Ec, 0.35), and pH (8.6).

The experiments were conducted on 2-year-old *Opuntia* spp. plants susceptible to *D. opuntiae* in a plot measuring 800 m^2^. The planting distance was 1 meter between rows and 0.5 meters between plants, resulting in approximately 12 rows with 19 plants per row, for a total of 228 plants in the plot. During the hot and dry season (June to September), each plant received 8 liters of water every 2 weeks. The annual water requirement was estimated at 30 liters per plant.

The plot was naturally infested with *D. opuntiae* and was surrounded by a hedge of *Opuntia* spp. already colonized by *D. opuntiae*. Additionally, all experimental plants were artificially infested by placing ten gravid females, obtained from the rearing system, on different cladodes of each plant. These females produced nymphs, which then spread naturally across the plants. The treatments were applied twenty days after the infestation with *D. opuntiae*.

Prior to this study, the plants were protected from damage by *D. opuntiae* using the same treatments applied in this study, but without the use of chemical products. The study was conducted from 2022 to 2024.

### 2.6 Treatments and experimental design

In all the three studied years, the following treatments were applied: T1 – untreated control, T2 – mineral oil (780 g/L) applied at 2400 cc/hL (MO), T3 – black soap applied at 60 g/L (BS), T4 – *N. glauca* at 10% (NG), T5 – *A. murispora* at 1.0 × 10^8^ conidia mL^−1^ (AM), T6 – MO + AM, T7 – BS + AM, T8 – NG + AM, T9 – MO + BS + NG and T10 – MO + BS + NG + AM. These treatments and concentrations were based on previous studies conducted under laboratory, greenhouse, and field conditions (El Aalaoui et al., 2019; Ramdani et al., 2021; Zim et al., 2024; El Aalaoui et al., 2024a). All treatments were applied with or without pruning. Pruning was not considered a standalone treatment; it was used as a supplementary practice to enhance the efficacy of the other treatments. Pruning was carried out annually before treatments application at the end of February, before the emission of floral and vegetative buds. Only overcrowded or competing cladodes were removed to ensure full coverage of all plant parts and maximize the effectiveness of the treatments.

The experiment followed a randomized complete block design (RCBD) with 19 treatments and 12 replicates (plants) for each treatment in each year. The treatments were applied using a Matabi sprayer (Super Green 16 L; Goizper S. Coop., Gipuzkoa, Spain) at a rate of 1,000 L/ha. For the combined treatments, the fungal suspension, plant extract, and/or botanical insecticide solution were mixed in equal proportions to achieve the desired volume. The mixtures were vortexed for 5 min to ensure uniformity.

The treatments were applied eight times per year. The first spraying was conducted at the beginning of March, followed by subsequent applications at 5-week intervals. To minimize spray drift to neighboring plants, polythene sheets were placed on the windward side.

### 2.7 Data collection and analysis

The evaluated variables included the number of fruits produced per plant (NF), the number of cladodes produced per plant (NCP), and fruit characteristics such as fruit weight (g) (FW) and fruit shape index (FSI), calculated as the ratio of fruit length (cm) to fruit diameter (cm).

The duration of the phenological phases of flowering and fruiting was also assessed. These phases included vegetative and floral bud emission (VFBE), flowering time (F), fruit maturation (FM), and the period of fruit development (PFD). The method described by Barbara (2007) was used to determine the duration of the flowering and fruiting phases.

The infestation level of *D. opuntiae* was scored using a grading scale: 0 (no colonies attached), 1 (up to 10 colonies per cladode), 2 (11–40 colonies per cladode), 3 (41–80 colonies per cladode), 4 (81–120 colonies per cladode), and 5 (more than 120 colonies per cladode) (de Vasconcelos et al., 2009).

Finally, the visual quality of treated plants was evaluated at the end of the experiment using a numerical scale from 0 to 10, as described by Gettys et al. (2021). On this scale, 0 represents dead plants, while 10 indicates excellent quality. This method has been widely used to assess plant responses to various factors, including herbicides, salt stress, and other environmental conditions (Gettys and Haller, 2012; Smith et al., 2014; Tootoonchi et al., 2020).

Data were analyzed using SPSS version 23. To assess the effects of treatment and time (year) separately, two one-way analyses of variance (ANOVA) were conducted. The first ANOVA evaluated the main effect of treatment on the response variables, while the second ANOVA assessed the main effect of time (year). Post-hoc comparisons were performed using the least significant difference (LSD) test to identify significant differences between treatments. Levene’s test for homogeneity of variance was used to verify the assumption of equal variances, and data were log-transformed if necessary to meet the assumptions of normality. Statistical significance was set at *α* = 0.05 and exact *p*-values are reported in the Results section. Results are presented as means ± standard error (SE).

## 3 Results

### 3.1 Effect of treatments on the number of fruits and cladodes produced per plant

The different treatments significantly (*p* < 0.05) affected the number of fruits per plant (NF) and the number of cladodes produced per plant (NCP) in *Opuntia* spp. across the 3 years of the study (2022–2024) (Table 1). The NF increased over time for all treatments, with the highest fruit and cladode production recorded in 2024.

**Table 1:** Effect of different treatments on the number of fruits per plant (NF) and the number of cladodes produced per plant (NCP) in treated *O. ficus-indica* plants.

Treatments	Number of fruits per plant (Mean± SE)			Number of cladodes initiated per plant (Mean± SE)		
	2022	2023	2024	2022	2023	2024
Untreated control	5.1 ± 0.6 ^IJa^	0.0 ± 0.0 ^Mb^	0.0 ± 0.0 ^Lb^	1.8 ± 1.2 ^HIa^	0.0 ± 0.0 ^Kb^	0.0 ± 0.0 ^Ib^
Mineral oil (MO)	14.0 ± 1.1 ^GHc^	36.6 ± 3.6 ^HIJKb^	64.7 ± 3.8 ^FGHIa^	5.8 ± 0.4 ^GHb^	16.6 ± 1.2 ^GHIb^	29.3 ± 2.4 ^Ga^
Black soap (BS)	14.1 ± 1.6 ^GHc^	50.6 ± 4.3 ^FGHb^	70.3 ± 3.7 ^EFGHa^	6.4 ± 0.7 ^Gc^	23.3 ± 2.5 ^FGb^	35.3 ± 2.4 ^FGa^
*Nicotiana glauca* (NG)	5.5 ± 0.7 ^IJc^	18.8 ± 3.4 ^KLb^	46.0 ± 3.7 ^JKa^	1.6 ± 1.2 ^HIc^	5.8 ± 0.9 ^JKb^	14.0 ± 0.8 ^Ha^
*Alternaria murispora* (AM)	4.5 ± 0.5 ^Jb^	13.3 ± 3.3 ^LMb^	40.9 ± 4.0 ^Ka^	1.4 ± 1.1 ^Ic^	4.5 ± 1.0 ^JKb^	12.2 ± 0.9 ^Ha^
MO + AM	20.5 ± 1.3 ^FGc^	43.3 ± 3.5 ^GHIb^	70.0 ± 3.9 ^EFGHa^	12.3 ± 0.8 ^Fc^	19.6 ± 1.7 ^FGHb^	33.8 ± 2.0 ^FGa^
BS + AM	27.5 ± 1.9 ^DEc^	70.7 ± 3.8 ^CDEb^	86.9 ± 3.0 ^CDEa^	18.0 ± 1.2 ^CDc^	36.9 ± 2.5 ^DEb^	47.8 ± 2.0 ^DEa^
NG + AM	9.6 ± 0.9 ^HIJc^	24.4 ± 3.4 ^JKLb^	51.0 ± 3.6 ^IJKa^	3.3 ± 0.3 ^GHIc^	8.3 ± 1.2 ^IJKb^	16.4 ± 1.1 ^Ha^
MO + BS + NG	39.9 ± 1.8 ^BCc^	80.7 ± 4.3 ^BCDb^	95.7 ± 3.2 ^BCDa^	17.4 ± 1.2 ^CDEc^	45.7 ± 3.0 ^CDb^	56.6 ± 2.2 ^CDa^
MO + BS + NG + AM	45.1 ± 1.9 ^ABc^	86.5 ± 4.5 ^ABCb^	102.3 ± 3.3 ^ABCa^	20.9 ± 1.3 ^BCc^	48.9 ± 3.1 ^BCb^	61.3 ± 2.3 ^BCa^
MO + pruning	20.0 ± 1.1 ^FGc^	52.7 ± 3.5 ^EFGHb^	75.8 ± 3.8 ^EFGa^	12.8 ± 0.8 ^Fc^	21.2 ± 1.7 ^FGHb^	39.9 ± 2.5 ^EFa^
BS + pruning	20.0 ± 1.6 ^FGc^	66.5 ± 4.3 ^DEFb^	81.2 ± 3.7 ^DEFa^	13.5 ± 1.2 ^EFc^	36.2 ± 2.8 ^DEb^	45.5 ± 2.6 ^Ea^
NG + pruning	11.5 ± 0.7 ^HIc^	34.8 ± 3.4 ^HIJKb^	57.0 ± 3.7 ^HIJKa^	4.3 ± 0.3 ^GHIc^	11.1 ± 0.8 ^HIJb^	14.0 ± 1.0 ^Ha^
AM+ pruning	10.4 ± 0.6 ^HIJc^	29.3 ± 3.3 ^IJKLb^	51.9 ± 4.0 ^IJKa^	3.7 ± 0.3 ^GHIc^	8.8 ± 0.8 ^IJKb^	13.8 ± 1.2 ^Ha^
MO + AM+ pruning	26.5 ± 1.3 ^EFc^	59.3 ± 3.5 ^EFGb^	81.0 ± 3.9 ^DEFa^	14.7 ± 0.8 ^DEFc^	28.7 ± 2.3 ^EFb^	42.9 ± 2.5 ^EFa^
BS + AM+ pruning	33.5 ± 1.8 ^CDc^	86.7 ± 3.8 ^ABCb^	97.9 ± 3.0 ^ABCa^	13.8 ± 0.9 ^DEFc^	49.8 ± 2.6 ^BCb^	57.4 ± 2.0 ^BCDa^
NG + AM+ pruning	15.6 ± 0.9 ^GHc^	40.4 ± 3.4 ^HIJb^	62.0 ± 3.6 ^GHIJa^	6.4 ± 0.5 ^Gc^	13.6 ± 0.8 ^GHIJb^	18.4 ± 1.3 ^Ha^
MO + BS + NG + pruning	45.9 ± 1.8 ^ABb^	96.7 ± 4.3 ^ABa^	106.7 ± 3.2 ^ABa^	22.7 ± 1.3 ^ABb^	59.2 ± 3.1 ^ABa^	66.6 ± 2.3 ^ABa^
MO + BS + NG + AM+ pruning	51.1 ± 1.9 ^Ab^	102.5 ± 4.5 ^Aa^	113.3 ± 3.2 ^Aa^	26.5 ± 1.4 ^Ab^	63.6 ± 3.2 ^Aa^	71.4 ± 2.3 ^Aa^

Means followed by the same capital letter within a column are not significantly different (LSD test, *p* < 0.05). Means followed by the same lowercase letter within a row are not significantly different (LSD test, *p* < 0.05).

Throughout the study, the combined treatments mineral oil (MO) + black soap (BS) + *N. glauca* (NG) + *A. murispora* (AM) and MO + BS + NG with pruning consistently resulted in the highest NF (2022: F_18,209_ = 115.8, *p* = 4.6 × 10^−98^; 2023: F_18,209_ = 63.9, *p* = 1.4 × 10^−74^; 2024: F _18,209_ = 62.1, *p* = 1.8 × 10^−73^) and NCP (2022: F_18,209_ = 82.7, *p* = 1.5 × 10^−84^; 2023: F_18,209_ = 86.3, *p* = 3.1 × 10^−86^; 2024: F_18,209_ = 119.9, *p* = 1.7 × 10^−99^). These treatments produced significantly more fruits and cladodes compared to all other treatments, including the untreated control.

The treatment MO + BS + NG + AM without pruning showed similarly high efficacy. It did not differ significantly in fruit production compared to MO + BS + NG + AM with pruning, but it ranked second in cladode production. Likewise, the combination BS + AM with pruning significantly enhanced both fruit yield and cladode production compared to the untreated control.

Among the individual treatments, BS and MO performed better than NG and AM alone. The lowest NF and NCP were observed in untreated control plants, followed by AM alone, which did not differ significantly from the untreated control in 2022 but showed a gradual increase in fruit and cladode production in subsequent years.

Pruning consistently improved the efficacy of all treatments, with combined treatments involving pruning yielding significantly higher NF and NCP values than their non-pruned counterparts.

### 3.2 Effect of treatments on fruit weight and shape index

The different treatments significantly (*p* < 0.05) influenced fruit weight (FW) and fruit shape index (FSI) of *Opuntia* spp. across the 3 years of the study (2022–2024) (Table 2). The combined treatments MO + BS + NG + AM + pruning, MO + BS + NG + pruning, BS + AM + pruning, MO + AM + pruning, MO + BS + NG + AM, and MO + BS + NG consistently produced the heaviest fruits (2022: F₁₈,₂₀₉ = 56.0, *p* = 1.3 × 10^−69^; 2023: F₁₈,₂₀₉ = 374.3, *p* = 3.8 × 10^−148^; 2024: F₁₈,₂₀₉ = 374.7, *p* = 3.4 × 10^−148^) and the highest fruit shape indices (2022: F₁₈,₂₀₉ = 56.0, *p* = 1.3 × 10^−69^; 2023: F₁₈,₂₀₉ = 374.3, *p* = 3.8 × 10^−148^; 2024: F₁₈,₂₀₉ = 374.7, *p* = 3.4 × 10^−148^), with significant differences among treatments. The highest FW and FSI values were recorded for the combined treatment MO + BS + NG + AM with pruning, reaching 192.7 g and 1.7 in 2024, respectively. This combination was significantly more effective than all other treatments, including its non-pruned counterpart, which ranked third after MO + BS + NG + pruning in FW and FSI across the 3 years. Among the individual treatments, BS and MO produced heavier fruits and higher FSI compared to NG and AM alone. The treatment MO consistently yielded fruits weighing between 172.3 and 175.2 g across the 3 years, with a consistent FSI of 1.6. Similarly, BS resulted in FW ranging from 175.3 to 178.4 g and an FSI of 1.6. NG and AM alone produced the smallest fruits and lowest FSI values, with no significant differences from the untreated control in 2022, but showing gradual improvements in subsequent years. Pruning consistently enhanced FW and FSI in all treatments. The combination MO + BS + NG + AM with pruning significantly outperformed the same combination without pruning, indicating the beneficial role of pruning in improving fruit quality. Furthermore, the combined treatments MO + BS + NG and BS + AM + pruning ranked fourth and fifth in FW and FSI, respectively, producing significantly heavier fruits compared to the untreated control and individual treatments.

**Table 2:** Effect of different treatments on fruit weight (g) (FW) and fruit shape index (FSI) in treated *O. ficus-indica* plants.

Treatments	Fruit weight (g) (Mean± SE)			Fruit shape index (Mean± SE)		
	2022 (*N* = 12)	2023 (*N* = 12)	2024 (*N* = 12)	2022 (*N* = 12)	2023 (*N* = 12)	2024 (*N* = 12)
Untreated control	143.2 ± 2.3 ^FGa^	0.0 ± 0.0 ^Hb^	0.0 ± 0.0 ^Hb^	1.3 ± 0.0 ^FGa^	0.0 ± 0.0 ^Hb^	0.0 ± 0.0 ^Hb^
Mineral oil (MO)	172.3 ± 1.6 ^Da^	173.2 ± 1.8 ^Da^	175.2 ± 1.7 ^Da^	1.6 ± 0.0 ^Da^	1.6 ± 0.0 ^Da^	1.6 ± 0.0 ^Da^
Black soap (BS)	175.3 ± 1.3 ^BCDa^	176.2 ± 1.1 ^CDa^	178.4 ± 1.1 ^CDa^	1.6 ± 0.0 ^BCDa^	1.6 ± 0.0 ^CDa^	1.6 ± 0.0 ^CDa^
*Nicotiana glauca* (NG)	146.8 ± 3.7 ^EFGa^	147.6 ± 3.7 ^EFGa^	148.8 ± 3.8 ^FGa^	1.3 ± 0.0 ^EFGa^	1.3 ± 0.0 ^EFGa^	1.3 ± 0.0 ^FGa^
*Alternaria murispora* (AM)	142.5 ± 3.6 ^Ga^	143.4 ± 3.6 ^Ga^	144.9 ± 3.7 ^Ga^	1.3 ± 0.0 ^Ga^	1.3 ± 0.0 ^Ga^	1.3 ± 0.0 ^Ga^
MO + AM	176.8 ± 1.5 ^BCDa^	177.8 ± 1.5 ^BCDa^	179.0 ± 1.6 ^BCDa^	1.6 ± 0.0 ^BCDa^	1.6 ± 0.0 ^BCDa^	1.6 ± 0.0 ^BCDa^
BS + AM	179.6 ± 1.3 ^ABCDa^	180.8 ± 1.3 ^ABCDa^	182.8 ± 1.5 ^ABCDa^	1.6 ± 0.0 ^ABCDa^	1.6 ± 0.0 ^ABCDa^	1.6 ± 0.0 ^ABCDa^
NG + AM	154.2 ± 2.9 ^EFa^	155.2 ± 2.9 ^EFa^	156.9 ± 2.9 ^EFa^	1.4 ± 0.0 ^EFa^	1.4 ± 0.0 ^EFa^	1.4 ± 0.0 ^EFa^
MO + BS + NG	182.7 ± 0.7 ^ABCDb^	184.3 ± 0.5 ^ABCb^	186.8 ± 0.6 ^ABCa^	1.6 ± 0.0 ^ABCDb^	1.7 ± 0.0 ^ABCb^	1.7 ± 0.0 ^ABCa^
MO + BS + NG + AM	185.0 ± 0.6 ^ABCb^	186.2 ± 0.4 ^ABCb^	188.7 ± 0.5 ^ABCa^	1.7 ± 0.0 ^ABCb^	1.7 ± 0.0 ^ABCb^	1.7 ± 0.0 ^ABCa^
MO + pruning	174.1 ± 1.8 ^CDa^	175.4 ± 2.0 ^CDa^	177.7 ± 2.0 ^CDa^	1.6 ± 0.0 ^CDa^	1.6 ± 0.0 ^CDa^	1.6 ± 0.0 ^CDa^
BS + pruning	176.7 ± 0.9 ^BCDb^	178.6 ± 1.2 ^BCDab^	181.1 ± 1.3 ^BCDa^	1.6 ± 0.0 ^BCDb^	1.6 ± 0.0 ^BCDab^	1.6 ± 0.0 ^BCDa^
NG + pruning	147.9 ± 3.8 ^EFGa^	149.3 ± 3.7 ^EFGa^	151.3 ± 3.6 ^EFGa^	1.3 ± 0.0 ^EFGa^	1.3 ± 0.0 ^EFGa^	1.4 ± 0.0 ^EFGa^
AM+ pruning	144.3 ± 3.5 ^FGa^	145.6 ± 3.4 ^FGa^	147.6 ± 3.4 ^FGa^	1.3 ± 0.0 ^FGa^	1.3 ± 0.0 ^FGa^	1.3 ± 0.0 ^FGa^
MO + AM+ pruning	179.5 ± 1.4 ^ABCDb^	181.5 ± 1.2 ^ABCDab^	184.0 ± 1.3 ^ABCDa^	1.6 ± 0.0 ^ABCDb^	1.6 ± 0.0 ^ABCDab^	1.7 ± 0.0 ^ABCDa^
BS + AM+ pruning	182.1 ± 1.6 ^ABCDa^	184.0 ± 1.9 ^ABCDa^	186.8 ± 1.9 ^ABCa^	1.6 ± 0.0 ^ABCDa^	1.7 ± 0.0 ^ABCDa^	1.7 ± 0.0 ^ABCa^
NG + AM+ pruning	156.0 ± 2.8 ^Ea^	158.1 ± 2.9 ^Ea^	160.3 ± 2.9 ^Ea^	1.4 ± 0.0 ^Ea^	1.4 ± 0.0 ^Ea^	1.4 ± 0.0 ^Ea^
MO + BS + NG + pruning	185.6 ± 0.9 ^ABb^	187.6 ± 1.2 ^ABab^	190.1 ± 1.4 ^ABa^	1.7 ± 0.0 ^ABb^	1.7 ± 0.0 ^ABab^	1.7 ± 0.0 ^ABa^
MO + BS + NG + AM+ pruning	188.2 ± 0.6 ^Ab^	189.9 ± 0.9 ^Aab^	192.7 ± 0.9 ^Aa^	1.7 ± 0.0 ^Ab^	1.7 ± 0.0 ^Aab^	1.7 ± 0.0 ^Aa^

Means followed by the same capital letter within a column are not significantly different (LSD test, *p* < 0.05). Means followed by the same lowercase letter within a row are not significantly different (LSD test, *p* < 0.05). ^N^Number of individuals used in the experiment.

### 3.3 Vegetative and floral bud emission and flowering time

The different treatments significantly (*p* < 0.05) influenced the duration of vegetative and floral bud emission (VFBE) and flowering time (F) of *Opuntia* spp. across the 3 years of the study (2022–2024) (Table 3). The combinations of MO, BS, NG, and AM with and without pruning, as well as MO + BS + NG + pruning, consistently resulted in the shortest VFBE (2022: F₁₈,₂₀₉ = 141.6, *p* = 2.2 × 10^−106^; 2023: F₁₈,₂₀₉ = 353.7, *p* = 1.1 × 10^−145^; 2024: F₁₈,₂₀₉ = 98.8, *p* = 1.3 × 10^−91^) and F durations (2022: F₁₈,₂₀₉ = 142.3, *p* = 1.4 × 10^−106^; 2023: F₁₈,₂₀₉ = 351.2, *p* = 2.4 × 10^−145^; 2024: F₁₈,₂₀₉ = 337.2, *p* = 1.4 × 10^−143^) across all study years. No significant differences were observed between these treatments and MO + BS + NG regarding VFBE and F in 2022, 2023, and 2024, and regarding F in 2022 and 2023. Additionally, no significant differences were detected between all the aforementioned treatments and BS + AM + pruning, MO + AM + pruning, BS + pruning, and BS + AM treatments regarding VFBE in 2024. Among the individual treatments, BS and MO treatments exhibited shorter VFBE and F durations compared to NG and AM alone treatments. NG and AM alone treatments resulted in the longest VFBE and F durations, with no significant differences from the untreated control in 2022. In 2023 and 2024, no vegetative and floral bud emission was observed in untreated plants. For all tested treatments, the VFBE and F durations decreased over time, with the shortest durations recorded in 2024. The combination treatments significantly reduced VFBE and F durations compared to individual treatments. Pruning further shortened VFBE and F durations across all treatments.

**Table 3:** Effect of different treatments on the duration of vegetative and floral bud emission (VFBE) and flowering time (F) in treated *O. ficus-indica* plants.

Treatments	Vegetative and floral bud emission (Days) (Mean± SE)			Flowering time (Days) (Mean± SE)		
	2022	2023	2024	2022	2023	2024
Untreated control	101.2 ± 1.5 ^ABa^	–	–	99.1 ± 1.5 ^ABa^	–	–
Mineral oil (MO)	80.3 ± 1.3 ^Ca^	76.7 ± 1.3 ^Ba^	76.2 ± 1.2 ^Ba^	77.7 ± 1.3 ^Ca^	75.6 ± 1.3 ^Ca^	74.8 ± 1.2 ^Ca^
Black soap (BS)	77.1 ± 1.2 ^CDa^	73.6 ± 1.4 ^BCa^	72.6 ± 1.4 ^BCa^	74.3 ± 1.2 ^CDa^	71.0 ± 1.0 ^CDab^	70.3 ± 1.0 ^CDb^
*Nicotiana glauca* (NG)	101.2 ± 1.5 ^ABa^	98.7 ± 1.5 ^Aa^	97.8 ± 1.5 ^Aa^	98.8 ± 1.5 ^ABa^	96.2 ± 1.6 ^ABa^	95.7 ± 1.6 ^ABa^
*Alternaria murispora* (AM)	104.5 ± 1.0 ^Aa^	101.8 ± 1.0 ^Aa^	101.1 ± 1.1 ^Aa^	101.5 ± 1.0 ^Aa^	98.6 ± 1.2 ^Aa^	98.2 ± 1.2 ^Aa^
MO + AM	78.1 ± 1.2 ^CDa^	75.4 ± 1.2 ^BCab^	73.6 ± 1.2 ^BCb^	75.1 ± 1.2 ^CDa^	72.5 ± 1.2 ^CDa^	71.8 ± 1.3 ^CDa^
BS + AM	74.9 ± 0.5 ^CDEa^	72.3 ± 0.6 ^BCDb^	70.3 ± 0.5 ^BCDc^	71.9 ± 0.5 ^CDEa^	69.5 ± 0.5 ^DEb^	68.8 ± 0.5 ^CDEb^
NG + AM	101.5 ± 1.6 ^ABa^	99.5 ± 1.5 ^Aa^	97.4 ± 1.6 ^Aa^	98.5 ± 1.6 ^ABa^	95.8 ± 1.6 ^ABa^	95.3 ± 1.6 ^ABa^
MO + BS + NG	69.9 ± 0.8 ^EFGa^	67.3 ± 0.9 ^DEFab^	65.3 ± 0.9 ^BCDb^	66.9 ± 0.8 ^EFGa^	64.5 ± 0.8 ^EFGab^	64.0 ± 0.8 ^EFb^
MO + BS + NG + AM	67.0 ± 1.2 ^FGa^	64.3 ± 1.3 ^EFab^	62.2 ± 1.4 ^CDb^	64.0 ± 1.2 ^FGa^	61.7 ± 1.2 ^FGa^	61.0 ± 1.2 ^FGa^
MO + pruning	77.8 ± 1.2 ^CDa^	75.1 ± 1.2 BCab	72.9 ± 1.4 ^BCb^	74.8 ± 1.2 ^CDa^	72.3 ± 1.3 ^CDa^	71.7 ± 1.3 ^CDa^
BS + pruning	74.3 ± 1.2 ^CDEa^	71.7 ± 1.2 ^BCDab^	69.3 ± 1.2 ^BCDb^	71.3 ± 1.2 ^DEa^	68.7 ± 1.2 ^DEa^	67.9 ± 1.2 ^DEa^
NG + pruning	97.9 ± 1.6 ^Ba^	96.0 ± 1.6 ^Aa^	94.5 ± 1.7 ^Aa^	94.9 ± 1.6 ^Ba^	92.8 ± 1.6 ^ABa^	92.3 ± 1.6 ^ABa^
AM+ pruning	101.3 ± 1.2 ^ABa^	99.3 ± 1.3 ^Aa^	106.0 ± 8.9 ^Aa^	98.3 ± 1.2 ^ABa^	96.0 ± 1.1 ^ABa^	95.4 ± 1.2 ^ABa^
MO + AM+ pruning	75.2 ± 1.1 ^CDEa^	73.2 ± 1.4 ^BCDab^	70.3 ± 1.2 ^BCDb^	72.2 ± 1.1 ^CDEa^	69.4 ± 1.0 ^DEa^	68.7 ± 1.0 ^DEa^
BS + AM+ pruning	72.4 ± 0.7 ^DEFa^	70.2 ± 0.7 ^CDEa^	67.6 ± 0.7 ^BCDb^	69.4 ± 0.7 ^DEFa^	66.7 ± 0.7 ^DEFb^	65.9 ± 0.7 ^DEFb^
NG + AM+ pruning	98.1 ± 1.5 ^Ba^	96.6 ± 1.8 ^Aa^	94.6 ± 1.4 ^Aa^	95.1 ± 1.5 ^Ba^	91.8 ± 2.1 ^Ba^	91.2 ± 2.1 ^Ba^
MO + BS + NG + pruning	67.0 ± 0.6 ^FGa^	64.4 ± 0.7 ^EFb^	62.2 ± 0.7 ^CDb^	64.0 ± 0.6 ^FGa^	61.1 ± 0.7 ^FGb^	60.3 ± 0.7 ^FGb^
MO + BS + NG + AM+ pruning	64.4 ± 1.2 ^Ga^	61.9 ± 1.3 ^Fab^	59.4 ± 1.3 ^Db^	61.4 ± 1.2 ^Ga^	58.6 ± 1.2 ^Ga^	57.8 ± 1.2 ^Ga^

Means followed by the same capital letter within a column are not significantly different (LSD test, *p* < 0.05). Means followed by the same lowercase letter within a row are not significantly different (LSD test, *p* < 0.05).

### 3.4 Fruit maturation and period of fruit development

The application of different treatments significantly affected fruit maturation (FM) and the period of fruit development (PFD) in treated *Opuntia* spp. plants across the 3 years of the study (2022–2024) (Table 4). The combination treatments MO + BS + NG + AM + pruning and MO + BS + NG, with and without pruning, consistently resulted in the shortest FM durations (2022: F₁₈,₂₀₉ = 88.2, *p* = 4.2 × 10^−87^; 2023: F₁₈,₂₀₉ = 378.0, *p* = 1.4 × 10^−148^; 2024: F₁₈,₂₀₉ = 347.7, *p* = 6.5 × 10^−145^) and PFD (2022: F₁₈,₂₀₉ = 141.6, *p* = 2.2 × 10^−106^; 2023: F₁₈,₂₀₉ = 464.8, *p* = 1.1 × 10^−157^; 2024: F₁₈,₂₀₉ = 436.7, *p* = 6.2 × 10^−155^), significantly differing from all other treatments. No significant difference was observed between these treatments and MO + BS + NG + AM regarding PFD duration in all studied years. Among the individual treatments, BS and MO significantly reduced FM and PFD compared to NG and AM alone. The longest FM and PFD durations were recorded in untreated control, NG, AM, and NG + AM treatments in 2022, and in NG, AM, and NG + AM treatments in 2023 and 2024. No fruit maturation or development was observed in untreated plants in 2023 and 2024, consistent with the absence of vegetative and floral bud emission. The combination treatments consistently outperformed individual treatments in reducing FM and PFD durations. Pruning further decreased FM and PFD durations, particularly when combined with MO and BS. Additionally, all treatments resulted in progressively shorter FM and PFD durations over the 3 years, with the shortest durations recorded in 2024.

**Table 4:** Effect of different treatments on fruit maturation (FM) and the period of fruit development (PFD) in treated *O. ficus-indica* plants.

Treatments	Fruit maturation (Days) (Mean± SE)			Period of fruit development (Days) (Mean± SE)		
	2022	2023	2024	2022	2023	2024
Untreated control	86.5 ± 0.7 ^Aa^	–	–	185.6 ± 2.1 ^Aa^	–	–
Mineral oil (MO)	74.3 ± 1.3 ^Ba^	73.5 ± 1.2 ^Ba^	72.3 ± 1.3 ^Ba^	152.0 ± 2.5 ^Ba^	149.1 ± 2.4 ^Ba^	147.2 ± 2.4 ^Ba^
Black soap (BS)	71.1 ± 1.2 ^BCDEa^	70.1 ± 1.2 ^BCDa^	69.1 ± 1.2 ^BCDEa^	145.4 ± 2.4 ^BCDa^	141.1 ± 2.0 ^BCDa^	139.3 ± 2.0 ^BCDa^
*Nicotiana glauca* (NG)	86.3 ± 1.0 ^Aa^	85.3 ± 1.0 ^Aa^	84.3 ± 1.0 ^Aa^	185.1 ± 2.2 ^Aa^	181.5 ± 2.3 ^Aa^	180.0 ± 2.3 ^Aa^
*Alternaria murispora* (AM)	87.3 ± 0.6 ^Aa^	86.3 ± 0.6 ^Aa^	85.3 ± 0.6 ^Aa^	188.8 ± 1.7 ^Aa^	184.8 ± 1.8 ^Aa^	183.4 ± 1.8 ^Aa^
MO + AM	72.1 ± 1.2 ^BCa^	71.1 ± 1.2 ^BCa^	70.1 ± 1.2 ^BCa^	147.2 ± 2.3 ^BCa^	143.6 ± 2.4 ^BCa^	141.8 ± 2.4 ^BCa^
BS + AM	68.9 ± 0.5 ^CDEFa^	67.9 ± 0.5 ^CDEab^	66.9 ± 0.5 ^CDEFGb^	140.8 ± 1.1 ^CDEa^	137.4 ± 1.1 ^CDab^	135.7 ± 1.0 ^CDEb^
NG + AM	87.5 ± 0.7 ^Aa^	86.5 ± 0.7 ^Aa^	85.5 ± 0.7 ^Aa^	186.0 ± 2.1 ^Aa^	182.3 ± 2.0 ^Aa^	180.8 ± 2.0 ^Aa^
MO + BS + NG	63.9 ± 0.8 ^FGHa^	63.2 ± 0.7 ^EFGa^	61.9 ± 0.8 ^GHa^	130.8 ± 1.5 ^EFGa^	127.7 ± 1.5 ^EFGa^	125.9 ± 1.6 ^EFGa^
MO + BS + NG + AM	66.8 ± 1.2 ^DEFGa^	65.8 ± 1.2 ^DEFGa^	64.8 ± 1.2 ^DEFGa^	130.8 ± 1.5 ^EFGa^	127.5 ± 1.5 ^EFGa^	125.8 ± 1.5 ^EFGa^
MO + pruning	71.8 ± 1.2 ^BCDa^	70.8 ± 1.2 ^BCa^	69.8 ± 1.2 ^BCDa^	146.7 ± 2.5 ^BCa^	143.1 ± 2.5 ^BCa^	141.5 ± 2.5 ^BCa^
BS + pruning	68.3 ± 1.2 ^CDEFGa^	67.3 ± 1.2 ^CDEFa^	66.3 ± 1.2 ^CDEFGa^	139.7 ± 2.4 ^CDEa^	136.0 ± 2.3 ^CDEa^	134.3 ± 2.3 ^CDEa^
NG + pruning	86.1 ± 0.6 ^Aa^	85.1 ± 0.6 ^Aa^	84.1 ± 0.6 ^Aa^	181.0 ± 1.9 ^Aa^	177.9 ± 1.9 ^Aa^	176.4 ± 1.9 ^Aa^
AM+ pruning	84.9 ± 1.0 ^Aa^	83.9 ± 1.0 ^Aa^	82.9 ± 1.0 ^Aa^	183.3 ± 2.2 ^Aa^	179.9 ± 2.1 ^Aa^	178.3 ± 2.1 ^Aa^
MO + AM+ pruning	69.2 ± 1.1 ^BCDEa^	68.2 ± 1.1 ^CDa^	67.2 ± 1.1 ^CDEFa^	141.3 ± 2.2 ^CDa^	137.6 ± 2.0 ^CDa^	135.8 ± 2.0 ^CDa^
BS + AM+ pruning	66.4 ± 0.7 ^EFGa^	65.4 ± 0.7 ^DEFGa^	64.4 ± 0.7 ^EFGa^	135.8 ± 1.3 ^DEFa^	132.1 ± 1.3 ^DEFab^	130.3 ± 1.4 ^DEFb^
NG + AM+ pruning	87.9 ± 1.1 ^Aa^	86.5 ± 1.0 ^Aa^	85.5 ± 1.0 ^Aa^	183.0 ± 2.3 ^Aa^	178.3 ± 2.5 ^Aa^	176.7 ± 2.6 ^Aa^
MO + BS + NG + pruning	63.5 ± 1.6 ^GHa^	62.8 ± 1.6 ^FGa^	62.3 ± 1.7 ^FGHa^	127.5 ± 1.7 ^FGa^	123.9 ± 1.7 ^FGa^	122.6 ± 1.6 ^FGa^
MO + BS + NG + AM+ pruning	60.9 ± 1.1 ^Ha^	61.7 ± 0.5 ^Ga^	59.3 ± 1.1 ^Ha^	122.3 ± 1.8 ^Ga^	120.3 ± 1.1 ^Ga^	117.1 ± 1.9 ^Ga^

Means followed by the same capital letter within a column are not significantly different (LSD test, *p* < 0.05). Means followed by the same lowercase letter within a row are not significantly different (LSD test, *p* < 0.05).

### 3.5 Incidence of *Dactylopius opuntiae* and visual quality of treated plants

**Figure 1:**
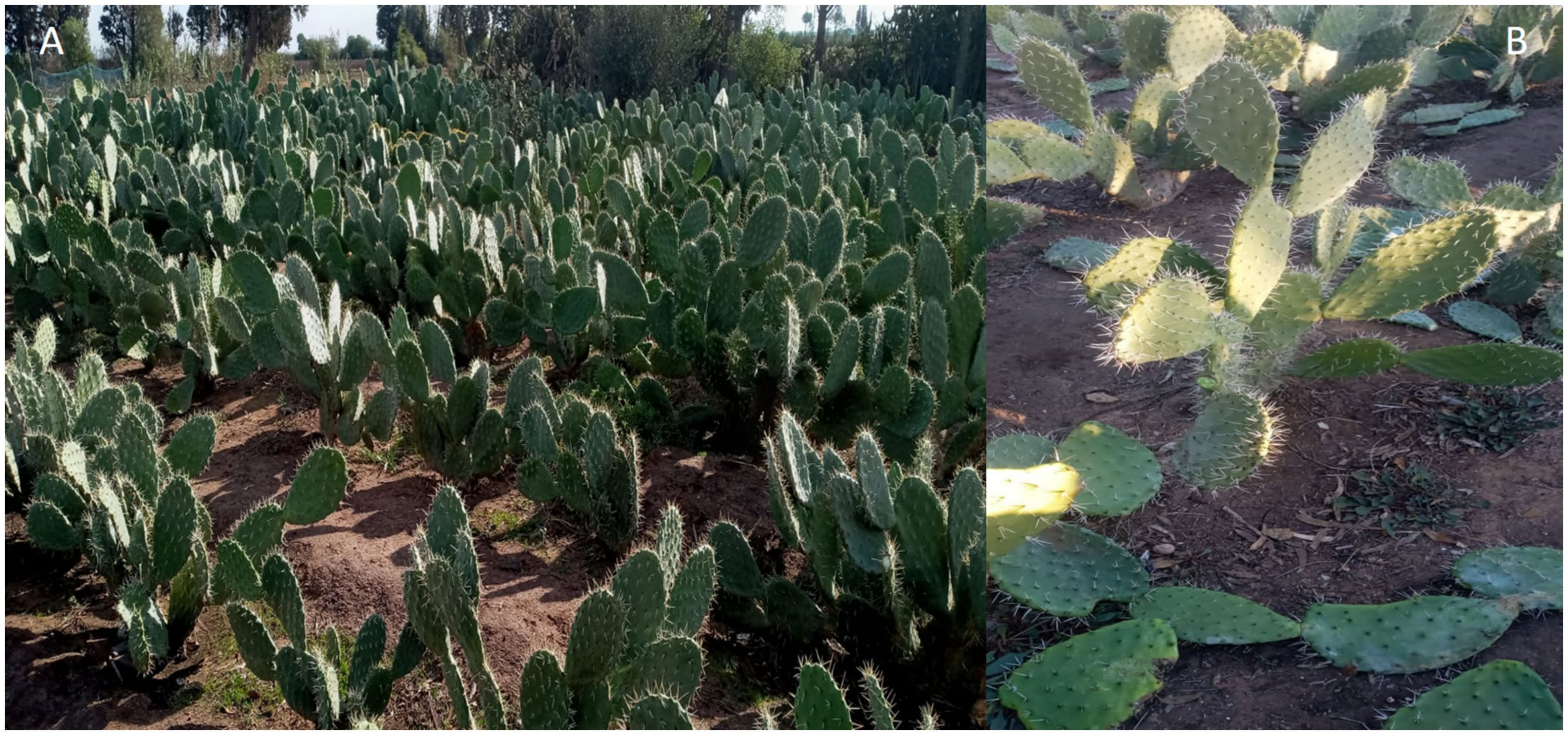
**(A)** *Opuntia* spp. plants from the experimental plot cultivated under integrated pest management strategies for *D. opuntiae*, including the combined use of biorational insecticides (mineral oil, black soap, and *Nicotiana glauca* extract) and the entomopathogenic fungus (*Alternaria murispora*), both alone and in combinations, with or without pruning. **(B)** Pruning of *Opuntia* spp. plants to enhance growth and pest control.

The results presented in Table 5 show the effects of different treatments on the incidence of *D. opuntiae* and the visual quality of treated *Opuntia* spp. plants across 3 years (2022–2024). The untreated control consistently exhibited the highest incidence of *D. opuntiae* (2022: F₁₈,₂₀₉ = 67.4, *p* = 1.3 × 10^−76^; 2023: F₁₈,₂₀₉ = 83.9, *p* = 4.2 × 10^−85^; 2024: F₁₈,₂₀₉ = 85.1, *p* = 1.1 × 10^−85^) and the lowest visual quality scores of treated plants (2022: F₁₈,₂₀₉ = 256.2, *p* = 1.3 × 10^−131^; 2023: F₁₈,₂₀₉ = 302.3, *p* = 8.5 × 10^−139^; 2024: F₁₈,₂₀₉ = 306.3, *p* = 2.3 × 10^−139^), compared to all tested treatments in all studied years. BS, MO, and NG treatments showed moderate reductions in *D. opuntiae* incidence and moderate visual quality scores of treated plants across all years. Combination treatments involving MO + BS + NG + AM + pruning, MO + BS + NG + pruning, BS + AM + pruning, MO + AM + pruning, BS + pruning, MO + pruning, MO + BS + NG + AM, MO + BS + NG, BS + AM, and MO + AM significantly reduced the incidence of *D. opuntiae* and enhanced the visual quality scores of treated plants in all years, with the lowest incidence and highest scores observed in 2024. Pruning combined with any treatment generally resulted in a further reduction of pest incidence and the highest visual quality scores (Figure 1). Overall, the combination of MO, BS, NG, and AM with or without pruning consistently outperformed individual treatments in reducing *D. opuntiae* incidence and enhancing the visual quality of treated plants, particularly in the later years.

**Table 5:** Effect of different treatments on the incidence of *D. opuntiae* and the visual quality of treated *O. ficus-indica* plants.

Treatments	Incidence of *D. opuntiae* (Mean± SE)			The visual quality of treated plants (1–10 scale) (Mean± SE)		
	2022	2023	2024	2022	2023	2024 F
Untreated control	5.0 ± 0.0 ^Aa^	5.0 ± 0.1 ^Aa^	5.0 ± 0.0 ^Aa^	1.0 ± 0.0 ^Ha^	0.8 ± 0.1 ^Hab^	0.6 ± 0.1^b^
Mineral oil (MO)	1.4 ± 0.1 ^BCDa^	1.2 ± 0.1 ^BCDa^	1.0 ± 0.2 ^BCDa^	8.6 ± 0.1 ^DEa^	8.8 ± 0.1 ^DEa^	9.0 ± 0.2 ^Ca^
Black soap (BS)	1.3 ± 0.1 ^BCDEa^	1.1 ± 0.1 ^BCDEa^	0.9 ± 0.1 ^BCDEa^	8.7 ± 0.1 ^CDEa^	8.9 ± 0.1 ^CDa^	9.1 ± 0.1 ^BCa^
*Nicotiana glauca* (NG)	1.5 ± 0.2 ^BCa^	1.3 ± 0.1 ^BCa^	1.3 ± 0.2 ^BCa^	6.5 ± 0.1 ^Ga^	6.7 ± 0.1 ^Ga^	6.8 ± 0.2 ^Ea^
*Alternaria murispora* (AM)	1.7 ± 0.1 B^a^	1.5 ± 0.2 ^Ba^	1.4 ± 0.2 ^Ba^	6.3 ± 0.1 ^Ga^	6.5 ± 0.2 ^Ga^	6.6 ± 0.2 ^Ea^
MO + AM	0.7 ± 0.1 ^FGHIa^	0.4 ± 0.1 ^FGHIa^	0.3 ± 0.1 ^FGa^	9.3 ± 0.1 ^ABa^	9.6 ± 0.1 ^ABa^	9.8 ± 0.1 ^Aa^
BS + AM	0.6 ± 0.1 ^FGHIa^	0.3 ± 0.1 ^GHIa^	0.2 ± 0.1 ^FGa^	9.4 ± 0.1 ^ABa^	9.7 ± 0.1 ^ABa^	9.8 ± 0.1 ^Aa^
NG + AM	0.9 ± 0.1 ^CDEFGa^	0.8 ± 0.1 ^CDEFGa^	0.7 ± 0.1 ^CDEFa^	8.1 ± 0.1 ^EFa^	8.3 ± 0.1 ^EFa^	8.3 ± 0.1 ^Da^
MO + BS + NG	0.4 ± 0.1 ^GHIa^	0.2 ± 0.1 ^GHIab^	0.0 ± 0.0 ^Gb^	9.6 ± 0.1 ^ABb^	9.8 ± 0.1 ^ABab^	10.0 ± 0.0 ^Aa^
MO + BS + NG + AM	0.3 ± 0.1 ^GHIa^	0.1 ± 0.1 ^HIab^	0.0 ± 0.0 ^Gb^	9.7 ± 0.1 ^ABb^	9.9 ± 0.1 ^ABab^	10.0 ± 0.0 ^Aa^
MO + pruning	0.8 ± 0.1^EFGHIa^	0.5 ± 0.2 ^EFGHIa^	0.3 ± 0.1 ^EFGa^	9.3 ± 0.1 ^ABCa^	9.5 ± 0.2 ^ABCa^	9.7 ± 0.1 ^ABa^
BS + pruning	0.7 ± 0.1 ^FGHIa^	0.4 ± 0.1 ^FGHIa^	0.3 ± 0.1 ^FGa^	9.3 ± 0.1 ^ABa^	9.6 ± 0.1 ^ABa^	9.8 ± 0.1 ^Aa^
NG + pruning	0.9 ± 0.1 ^CDEFGa^	0.8 ± 0.1 ^CDEFGa^	0.7 ± 0.1 ^CDEFa^	8.1 ± 0.1 ^EFa^	8.3 ± 0.1 ^EFa^	8.3 ± 0.1 ^Da^
AM+ pruning	1.7 ± 0.1^BCDEFa^	1.0 ± 0.2 ^BCDEFa^	0.9 ± 0.2 ^BCDEa^	7.8 ± 0.1 ^Fa^	8.0 ± 0.2 ^Fa^	8.1 ± 0.2 ^Da^
MO + AM+ pruning	0.3 ± 0.1 ^GHIa^	0.1 ± 0.1 ^HIab^	0.0 ± 0.0 ^Gb^	9.7 ± 0.1 ^ABb^	9.9 ± 0.1 ^ABab^	10.0 ± 0.2 ^Aa^
BS + AM+ pruning	0.4 ± 0.1 ^GHIa^	0.2 ± 0.1 ^GHIab^	0.0 ± 0.0 ^Gb^	9.6 ± 0.1 ^ABb^	9.8 ± 0.1 ^ABab^	10.0 ± 0.0 ^Aa^
NG + AM+ pruning	0.8 ± 0.1 ^DEFGHa^	0.7 ± 0.1 ^DEFGHa^	0.6 ± 0.1 ^DEFGa^	9.2 ± 0.1 ^BCDa^	9.3 ± 0.1 ^BCDa^	9.4 ± 0.1 ^ABCa^
MO + BS + NG + pruning	0.3 ± 0.1 ^HIa^	0.1 ± 0.1 ^HIa^	0.0 ± 0.0 ^Ga^	9.8 ± 0.1 ^ABa^	9.9 ± 0.1 ^ABa^	10.0 ± 0.0 ^Aa^
MO + BS + NG + AM+ pruning	0.2 ± 0.1 ^Ia^	0.0 ± 0.0 ^Ia^	0.0 ± 0.0 ^Ga^	9.8 ± 0.1 ^Aa^	10.0 ± 0.0 ^Aa^	10.0 ± 0.0 ^Aa^

Means followed by the same capital letter within a column are not significantly different (LSD test, *p* < 0.05). Means followed by the same lowercase letter within a row are not significantly different (LSD test, *p* < 0.05).

## 4 Discussion

Each of the tested control methods produced varying effects on *D. opuntiae* infestation, the visual quality of treated plants, and plant productivity parameters. These parameters included the number of fruits and cladodes per plant, fruit weight and shape index, vegetative and floral bud emission, flowering time, fruit maturation, and the period of fruit development. Among the treatments, the combined methods of mineral oil (780 g/L) at 2400 cc/hL (MO) + *N. glauca* at 10% (NG) + pruning and MO + BS + NG + *A. murispora* at 1.0 × 10^8^ conidia mL^−1^ (AM) + pruning were the most effective in reducing *D. opuntiae* incidence. These treatments maintained high visual quality of the treated plants. They consistently resulted in the highest fruit and cladode production, fruit weight, and shape index. Additionally, these combined treatments promoted faster vegetative and floral bud emission, earlier flowering, fruit maturation, and a shorter period of fruit development compared to the individual treatments and the untreated control.

The enhanced performance of the combined treatments (MO + BS + NG + pruning and MO + BS + NG + AM + pruning) can be attributed to the additive or synergistic effects of biorational insecticides (mineral oil and black soap), plant extracts, and entomopathogenic fungi.

Mineral oil and black soap reduce *D. opuntiae* populations through physical and biochemical mechanisms such as suffocation and disruption of insect cuticles (El Aalaoui et al., 2019; Ramdani et al., 2021). Mineral oil blocks the spiracles of both adult and nymphal pests, thereby preventing gas exchange in eggs, which ultimately causing asphyxiation and death (Cranshaw and Baxendale, 2011; Helmy et al., 2012). It is widely used in agriculture to control various pests on different crops (Agnello, 2001; El Aalaoui et al., 2019). Several studies have demonstrated the ovicidal activity of mineral oil (Riedl et al., 1995; El Aalaoui et al., 2019), and in some cases, it may also prevent oviposition (Riedl et al., 1995; Fernandez et al., 2001). Additionally, mineral oil has been shown to reduce both the number of eggs laid and the survival rate of insects pest eggs (Wins-Purdy et al., 2009).

Moroccan black soap is a traditional product made from natural fatty acids derived from olive oil. Soaps and oils are among the oldest chemicals used for insect control (Puritch, 1981). Several studies have reported the high insecticidal efficacy of mineral oil and soap against various insect pests, particularly scale insects, aphids, thrips, and mites (Wins-Purdy et al., 2009; de Souza Born et al., 2009; Curkovic, 2016). These effects were generally observed at the highest tested concentrations, typically at or below 2%, with repeated applications (Curkovic, 2016).

The addition of *N. glauca* extract, a plant extract with insecticidal properties, likely improved the efficacy of these treatments, as reported in previous studies (Ramdani et al., 2021; Zim et al., 2024). *N. glauca* has demonstrated insecticidal properties against various pests under both laboratory and field conditions, including the red palm weevil *Rhynchophorus ferrugineus* (Olivier) (Coleoptera: Curculionidae) (Ali Alghamdi, 2021), *P. solenopsis* (Abdelgader and Elawad, 2022), and *D. opuntiae* (Zim et al., 2024).

The addition of *A. murispora* in the MO + BS + NG + AM + pruning treatment further enhanced its efficacy, making it the most effective treatment among the tested methods in terms of pest control as well as fruit and cladode production and plant vigor. The higher greenhouse and field efficacy of *A. murispora* against *D. opuntiae* was also observed by El Aalaoui et al. (2024a).

Alternaria isolates, including *A. destruens* (Pleosporales: Pleosporaceae), have shown potential in controlling various insect pests. *Alternaria destruens* AKL-3 (Fr.) (Pleosporales: Pleosporaceae) significantly reduces consumption and growth in *Spodoptera litura* Fabricius (Lepidoptera: Noctuidae) larvae (Kaur et al., 2019). *Alternaria alternata* (Fr.) Keissl. has demonstrated efficacy against many insect pests, including *Oulema gallaeciana* Heyden (Coleoptera: Chrysomelidae), and *Zyginidia pullula* Boheman (Hemiptera: Cicadellidae) (Sharma and Sharma, 2014). In field experiments, *A. alternata* showed 81.14% mortality in *Myzus persicae* Sulzer (Hemiptera: Aphididae) and 63.14% mortality in *Lipaphis erysimi* Kaltenbach (Hemiptera: Aphididae), with no harmful effects on *Apis mellifera* Linnaeus (Hymenoptera: Apidae) and *Coccinella septempunctata* Linnaeus (Coleoptera: Coccinellidae) (Paschapur et al., 2022).

Both *A. destruens* and *A. murispora* produce proteases and chitinases that enhance their insecticidal properties (Green et al., 2001; El Aalaoui et al., 2024a). These fungi complete their life cycle within 48–72 h, causing brownish to blackish discoloration of infected insects, which become sluggish, stop feeding, and develop extensive mycelial growth (Christias et al., 2001). Their endophytic behavior allows spores to persist in plant tissues for months, ensuring pest control through ingestion of spore-colonized tissues (Allegrucci et al., 2017). This ability helps the fungus survive unfavorable environmental conditions (Klieber and Reineke, 2016). Accurate identification of fungal isolates is essential to optimize their effectiveness in biological control applications (Carneiro-Leão et al., 2017).

The highest effectiveness of the MO + BS + NG + AM + pruning treatment can also be explained by the lipophilic properties of mineral oil, which enable it to adhere strongly to the hydrophobic surfaces of fungal conidia and insect cuticles, enhancing its role as a spray carrier and sticker (Wraight et al., 2016). Oils spread quickly across insect cuticles, potentially transporting conidia into protected areas of the insect body, such as intersegmental regions, where moisture levels may favor germination and infection (Luz et al., 2012; Gospodarek et al., 2025).

Pruning enhanced the effectiveness of treatments by removing heavily infested cladodes, improving plant vigor, and facilitating treatment penetration. This effect was especially notable in combined treatments, which showed increased fruit and cladode numbers, as well as fruit weight and shape index, compared to non-pruned plants. Improved plant health likely contributed to shorter vegetative and floral bud emission periods, earlier flowering, and more synchronized fruit set (Milivojevic et al., 2025). These findings align with previous studies that show healthier plants tend to flower and fruit earlier under optimal conditions (Asin et al., 2007; Holb et al., 2010; Brenard et al., 2020).

Fruit maturation and the fruit development period were significantly shorter in plants treated with combined methods, particularly those involving pruning. This outcome suggests that the applied treatments accelerated plant development, likely due to the combined effects of pest suppression, improved plant vigor, and enhanced resource allocation. In line with this, Biondi et al. (2018) also reported reduced tomato crop yields due to damage by *Tuta absoluta* Meyrick (Lepidoptera: Gelechiidae) and emphasized the importance of carefully selecting and implementing effective control measures against this pest to avoid yield losses, which can reach up to 100%. The gradual improvements observed in NG and AM treatments throughout the study period indicate that these methods may require longer application periods to reach their full potential.

Spring pruning at the end of February, before vegetative and floral bud emission, could be an effective strategy for controlling *D. opuntiae*. However, it should not be the sole method used for controlling insect infestations and promoting plant growth. The combination of MO, BS, NG, and AM with pruning presents a promising sustainable approach for managing *D. opuntiae* infestations while improving the yield and quality of *Opuntia* spp. Over the 3 years of the study, this approach provided effective control and resulted in the highest yield.

## 5 Conclusion

This study demonstrates that integrated pest management (IPM) strategies combining pruning, biorational insecticides (mineral oil, black soap), plant extracts (*N. glauca*), and entomopathogenic fungi (*A. murispora*) can effectively enhance the growth, development, and health of *O. ficus-indica* while suppressing *D. opuntiae* populations. Among the tested approaches, combined treatments consistently provided the greatest benefits, highlighting the importance of integrating multiple control methods. Pruning was particularly important for optimizing pest suppression and plant productivity. These findings indicate that IPM strategies can offer sustainable and environmentally friendly alternatives to chemical control, but their practical implementation requires careful planning and optimization. Future research should focus on refining treatment combinations, timing, and dosages, and on validating long-term effectiveness and scalability under diverse field conditions.

## Data Availability

The original contributions presented in the study are included in the article/supplementary material, further inquiries can be directed to the corresponding author.
